# Incomplete *Plasmodium falciparum* growth inhibition following piperaquine treatment translates into increased parasite viability in the *in vitro* parasite reduction ratio assay

**DOI:** 10.3389/fcimb.2024.1396786

**Published:** 2024-04-30

**Authors:** Annabelle Walz, Sibylle Sax, Christian Scheurer, Balint Tamasi, Pascal Mäser, Sergio Wittlin

**Affiliations:** ^1^ Department of Medical Parasitology and Infection Biology, Swiss Tropical and Public Health Institute, Allschwil, Switzerland; ^2^ University of Basel, Basel, Switzerland

**Keywords:** PRR assay, drug resistance, piperaquine, parasite viability, growth inhibition assay, 4-aminoquinoline

## Abstract

Antimalarial resistance to the first-line partner drug piperaquine (PPQ) threatens the effectiveness of artemisinin-based combination therapy. *In vitro* piperaquine resistance is characterized by incomplete growth inhibition, i.e. increased parasite growth at higher drug concentrations. However, the 50% inhibitory concentrations (IC_50_) remain relatively stable across parasite lines. Measuring parasite viability of a drug-resistant Cambodian *Plasmodium falciparum* isolate in a parasite reduction ratio (PRR) assay helped to better understand the resistance phenotype towards PPQ. In this parasite isolate, incomplete growth inhibition translated to only a 2.5-fold increase in IC_50_ but a dramatic decrease of parasite killing in the PRR assay. Hence, this pilot study reveals the potential of *in vitro* parasite viability assays as an important, additional tool when it comes to guiding decision-making in preclinical drug development and post approval. To the best of our knowledge, this is the first time that a compound was tested against a drug-resistant parasite in the *in vitro* PRR assay.

## 1 Introduction

Artemisinin-based combination therapies (ACTs) are the mainstay of malaria therapy. Since their introduction about 20 years ago, they have prevented the death of millions (World Health Organization, 2023). Today, resistance to artemisinin and its partner drugs is threatening the effectiveness of ACTs (World Health Organization, 2023). Indeed, reports of ACT treatment failures are mounting. Amongst the six ACTs currently recommended by the World Health Organization for the treatment of uncomplicated malaria (World Health Organization, 2022), dihydroartemisinin-piperaquine (DHA-PPQ) experiences most treatment failures. For instance, half of the therapeutic efficacy studies conducted in the WHO Western Pacific Region between 2015 and 2021 reported treatment failure in at least 10% of the participants following DHA-PPQ therapy (World Health Organization, 2023).

Whilst reduced susceptibility to DHA (or artemisinin) is well described (Teuscher et al., 2010; Witkowski et al., 2010; Witkowski et al., 2013; Ariey et al., 2014; Birnbaum et al., 2020), the mechanism of PPQ resistance is not fully elucidated yet. Elevated 50% and 90% inhibitory concentrations (IC_50_ and IC_90_) of PPQ immediately determined *ex vivo* in Cambodian patient isolates were detected as early as 2013 and showed a significant association with DHA-PPQ treatment failure (Saunders et al., 2015; Chaorattanakawee et al., 2016). In addition, culture-adapted, *k13*-mutant parasites isolated from DHA-PPQ-treated patients in Cambodia repeatedly demonstrated abnormal dose-response curves in *in vitro* growth inhibition assays following PPQ exposure (Duru et al., 2015; Bopp et al., 2018; Ross et al., 2018). These curves were characterized by persistent parasite growth, i.e. incomplete growth inhibition, or even an increase in parasite growth at high drug concentrations (~200 nM and higher), and hence were often difficult to interpret. To allow for better monitoring of PPQ resistance, Duru et al. developed the PPQ survival assay (PSA_0-3h_), a washout assay tailored to the pharmacological profile of the drug in humans (Duru et al., 2015). Increased survival in the PSA_0-3h_ (i.e. > 10%) was not only associated with abnormal dose-response curves, but also with treatment failure in the patients from whom the parasite isolate originated (Duru et al., 2015). Finally, studies aiming at identifying genetic markers of PPQ resistance pointed to an association with amplifications in *plasmepsin 2* and *3* (Agrawal et al., 2017; Amato et al., 2017; Witkowski et al., 2017; Bopp et al., 2018; Kane et al., 2023), or single nucleotide polymorphisms in *exonuclease* (Amato et al., 2017) and *chloroquine-resistance transporter* (*crt*) (Duru et al., 2015; Agrawal et al., 2017; Dhingra et al., 2017; Ross et al., 2018; Dhingra et al., 2019; Gomez et al., 2023; Kane et al., 2023).

Compounds exhibiting an incomplete growth inhibition phenotype are routinely being picked up in drug screening activities conducted on a panel of drug-resistant field isolates, e.g. as observed in a lead-optimization study on 2,6-imidazopyridines (Le Manach et al., 2018). In fact, this phenotype is considered a warning sign and often prompts closer examination. In this pilot study, we were interested in how a compound with an incomplete growth inhibition phenotype, i.e. PPQ, performs when measuring parasite viability rather parasite growth. Providing highly sensitive and rich pharmacodynamic data, parasite viability has proven to be a superior measure of drug activity than conventional readouts such as *in vitro* growth inhibition or *in vivo* parasite clearance (Sanz et al., 2012; Rebelo et al., 2020; Walz et al., 2023). Therefore, we tested PPQ in the *in vitro* parasite reduction ratio (PRR) assay version 2 (Walz et al., 2023), an assay used to measure parasite viability, against the drug-sensitive *P. falciparum* strain NF54 and the Cambodian field isolate RF12 (also known in the literature as PH1263-C (Ross et al., 2018)). We show that measuring parasite viability provides a much clearer picture of the resistance phenotype to PPQ, and hence might also have the potential to support decision-making with respect to candidate prioritization in preclinical development and treatment policy changes in the post-marketing phase.

## 2 Materials and methods

### 2.1 Compounds

Piperaquine tetraphosphate (piperaquine, PPQ) powder was obtained from AK Scientific (#H853, Lot 70313H, 98% purity) and has a molecular weight of 999.55 g/mol.

Chloroquine diphosphate (CQ) powder served as an internal control in the growth inhibition assays. It was purchased from Sigma Life Science (#C6628, Lot BCBM9716V, ≥ 98% purity) and has a molecular weight of 515.86 g/mol.

### 2.2 Parasite origin and cultivation

The drug-sensitive *Plasmodium falciparum* strain NF54 (isolated from a patient living close to an airport in the Netherlands) was kindly provided by F. Hoffmann-La Roche Ltd. (Basel, Switzerland). The Cambodian patient isolate PH1263-C (RF12) was a gift from Prof. Dr. David Fidock (Columbia University Irving Medical Center, New York), harbors a H97Y and a C580Y mutation on the *P. falciparum chloroquine resistance transporter* gene and the *kelch 13* gene, respectively, and carries a single copy of the *P. falciparum multidrug resistance 1* gene (Ross et al., 2018). Asexual blood stages of both strains were maintained in humidified modular chambers at 37°C and 93% N_2_, 4% CO_2_, and 3% O_2_ (hereinafter referred to as “standard conditions”) in accordance with Snyder et al. (2007). The culture medium (CM) consisted of RPMI 1640 (10.44 g/L) supplemented with HEPES (5.94 g/L), NaHCO_3_ (2.1 g/L), Neomycin (100 μg/mL), hypoxanthine (50 mg/L), and albuMAX™ (5 g/L). The human erythrocytes were obtained from the blood donation center Zurich.

### 2.3 [^3^H]hypoxanthine growth inhibition assay

Inhibition of parasite growth was assessed via the incorporation of radiolabeled hypoxanthine and in accordance with Snyder et al. (2007). Briefly, unsynchronized *P. falciparum* cultures (NF54 or RF12) were exposed to a 64-fold range of compound at a parasitemia of 0.3% and a hematocrit of 1.25% under standard conditions. After 48 hours, 0.25 µCi of [^3^H]hypoxanthine was added and the cultures were incubated for another 24 hours. The assay was terminated by fully freezing the culture plates at -20°C. Thawed plates were harvested with a Microbeta FilterMate cell harvester (Perkin Elmer, Waltham, MA, USA), which transferred the lysed red blood cells onto a glass fiber filter. The dried filters were inserted into a plastic foil with 3.5 mL of scintillation fluid and counted in a MicroBeta2 liquid scintillation counter (Perkin Elmer, Waltham, MA, USA). The results were recorded as counts per minute (cpm) and individual IC_50_ values were calculated by linear interpolation (Huber and Koella, 1993) in a graphical program. CQ served as internal compound control and the obtained IC_50_ values were in alignment with our own previously published data (Walz et al., 2023).

To calculate median IC_50_ values, we estimated Bayesian hierarchical dose-response models for each strain-drug combination separately. Within each model, the hierarchical model structure allows the individual biological replicates to differ in their dose-response characteristics, but still assumes that there exists a “population level” dose-response relationship of which the individual biological replicates represent special cases. With this modeling approach, we retained flexibility in terms of between-group (i.e., on the level of biological replicates) variations, but also achieve partial pooling, that is, we borrow information from all observations for the estimation of each single, replicate-level dose-response parameter. Moreover, as the hierarchical model naturally accommodates grouped data, our inference naturally takes the correlation structure in the dataset into account. The Bayesian estimation with Markov Chain Monte Carlo (Carpenter et al., 2017) enables us to perform exact inference on the population-level dose-response curves, which is of primary interest in our analysis. Detailed description of the statistical model is given in the **Statistical Appendix** (Section 1), along with considerations regarding the error structure of the model (Section 1.3), the different model variants (Sections 1.1 and 1.3), prior choices (Section 1.2), and model checking results (Sections 2.4 and 2.5).

### 2.4 Parasite reduction ratio assay

Viability of the parasites was assessed according to the PRR assay V2 (Walz et al., 2023). In brief, unsynchronized *P. falciparum* cultures (NF54 and RF12, with average growth rates (ln scale) ranging from 0.047 to 0.048 and from 0.041 to 0.043, respectively (**Supplementary Figure S1**)) were adjusted to 0.3% parasitemia and 1.25% hematocrit using fresh human erythrocytes and CM. To initiate the assay, culture aliquots were incubated in 6-well plates (Falcon #353046) with fresh compound solution at a concentration corresponding to 10 × IC_50_ and under standard conditions. CM and compound were replenished every 24 hours. Before the first treatment (0 hours) and after 24, 48, 72, 96 and 120 hours, 3 mL of culture were sampled from the corresponding well and compound was removed by washing three times in 3 mL of CM (centrifugation: 2 min, 600 g). The complete removal of compound after washing was verified by incubating the supernatant recovered after the last washing step with fresh cultures of parasites for 72 h, ensuring that no growth inhibition was detected. In a 96-well plate (Sarstedt #83.3924), four technical replicates of each sample (eight for untreated controls) were serially diluted by factor four before being incubated again for 14 days. Once a week, culture medium was replenished and fresh erythrocytes were provided. After 13 days, the medium was replaced with 0.5 µCi of [^3^H]hypoxanthine in hypoxanthine-free CM and another 24 hours later, the plates were put at -20°C until fully frozen. Thawed plates were harvested with a Microbeta FilterMate cell harvester (Perkin Elmer, Waltham, MA, USA), which transferred the lysed red blood cells onto a glass fiber filter. The dried filters were inserted into a plastic foil with 3.5 mL of scintillation fluid and counted in a MicroBeta2 liquid scintillation counter (Perkin Elmer, Waltham, MA, USA). The results were recorded as cpm. In addition, colored spots on the dry filter mat were recorded. They served as visual indicator for parasite growth. Untreated cultures (0 and 48 hours incubation, microscopic readout) served as growth controls. Data analysis was conducted in R (version 4.1.3) and RStudio (version 2022.02.3) according to Walz et al. (2023) unless a compound was inactive (i.e. no reduction in viable parasites even after 120 hours of compound exposure). For inactive compounds, all parameters were defined as “NA” or “> 120” [hours].

## 3 Results

**Figure 1:**
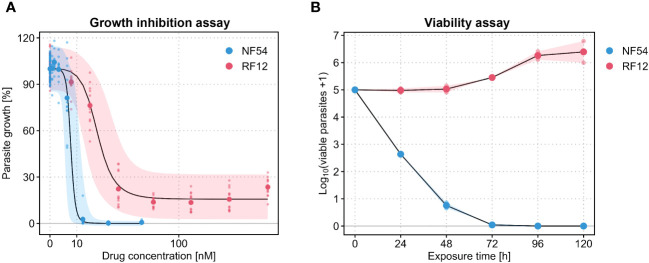
**(A)** Piperaquine (PPQ) dose-response curves generated in a [^3^H]hypoxanthine growth inhibition assay with *P. falciparum* NF54 (blue) and RF12 (red). Large dots are the means of n = 6 biological replicates, each consisting of n = 2 technical replicates (small dots). The black lines are the medians of the posterior predictive distribution and the shaded areas denote the 90% predictive intervals. **(B)** PPQ killing curves generated in parasite reduction ratio assays with the same *P. falciparum* isolates. Large dots are the means of n = 2 biological replicates and small dots are the means of n = 4 technical replicates from within each biological replicate. The shaded area represents the range between the two biological replicates. The killing curves are based on a PPQ concentration corresponding to 10× IC_50_ of the respective *P. falciparum* isolate.

We first wanted to reproduce the abnormal dose-response curves of PPQ published by Ross et al. (2018). For this, we ran growth inhibition assays with the pan-sensitive *P. falciparum* isolate NF54 and the PPQ-resistant Cambodian field isolate RF12, which carries mutations in *kelch13* (C580Y) and *Pfcrt* (H97Y) and a single copy of *Pfmdr1.* Both lines were tested in n = 6 biological replicates (each with n = 2 technical replicates). Whilst the sensitive parasite isolate displayed an exemplary sigmoidal dose-response curve with a single-digit nanomolar IC_50_ value (7.7 nM) for PPQ (**Table 1**), the Cambodian isolate presented an abnormal dose-response phenotype that was characterized by a plateau at around 15.3% parasite growth towards the highest PPQ concentrations tested, i.e. an incomplete growth inhibition phenotype (**Figure 1A**). The median IC_50_ of the Cambodian isolate increased by a factor of 2.5 to 19.3 nM compared to the drug-sensitive parasite isolate (**Table 1**).

**Table 1:** Pharmacodynamic parameters of piperaquine (PPQ) generated in the growth inhibition assay and the viability (PRR) assay with *P. falciparum* NF54 and RF12.

	*P. falciparum* NF54	*P. falciparum* RF12
Growth inhibition assay		
Median IC_50_ [90% credible interval] (nM)	7.66 [5.30 – 11.43]	19.25 [12.47 – 29.65]
Hill slope [90% credible interval]	9.28 [6.21 – 16.84]	4.98 [3.36 – 8.39]
% parasite growth at plateau	0	15.31 [7.6 – 28.09]
Viability (PRR) assay		
Log_10_(PRR) [min, max]	4.4 [4.3, 4.4]	NA
Lag time [min, max] (h)	0 [0, 0]	>120
PCT_99.9%_ [min, max] (h)	33.2 [32.5, 33.9]	>120
Pharmacodynamic category	fast	slow/inactive

The PRR assay parameters are based on an assay concentration corresponding to 10× IC_50_ of the respective *P. falciparum* isolate. For logistical reasons, this average IC_50_ value was calculated from a subset of the n = 6 biological replicates used in the dose-response analysis of the growth inhibition assay. IC_50_, 50% inhibitory concentration; PRR, parasite reduction ratio assay; PCT_99.9%_, 99.9% parasite clearance time. NA, not applicable.

In a next step, we used the same two parasite isolates to initiate independent PRR assays in order to assess parasite viability following PPQ exposure. In contrast to the growth inhibition assay, here the parasites are serially diluted and re-cultivated in the absence of drug after a certain duration of drug exposure, so that they – if viable – can reestablish a parasite culture. In line with previous experiments conducted in-house with the drug-sensitive *P. falciparum* isolate NF54, PPQ treatment resulted in a fast, chloroquine-like killing profile. An immediate onset of drug activity was accompanied by a PRR of 4.4 rendering almost all parasites non-viable after 48 hours of drug exposure (**Figure 1B**, **Table 1**). In contrast, when assessed in the Cambodian isolate RF12, PPQ treatment did not result in a decline of viable parasites (**Figure 1B**). Instead, the number of viable parasites increased by a factor of 10, approximately, within the first 96 hours of treatment, hence indicating a lack of drug activity in this parasite isolate.

## 4 Discussion

In the recent years, field isolates of *P. falciparum* have repeatedly demonstrated abnormal dose-response curves following PPQ exposure *in vitro*. Stagnation or even increase of parasite growth towards higher PPQ concentrations was associated with increased survival in the PSA (Duru et al., 2015; Ross et al., 2018), specific genotypes (Bopp et al., 2018; Ross et al., 2018), and with increased treatment failure in DHA-PPQ-treated patients (Duru et al., 2015). With the goal to better understand this incomplete growth inhibition phenotype, we assessed the viability of a culture-adapted field isolate in the PRR assay, i.e. a viability assay. To the best of our knowledge, this is the first time a *P. falciparum* strain other than the drug-sensitive ones (*P. falciparum* 3D7 or NF54) was tested in the *in vitro* PRR assay. Using PPQ as an example, we found that incomplete growth inhibition translates in a drastic increase in parasite viability when assessed in a PPQ-resistant Cambodian field isolate as compared to its drug-sensitive counterpart. Importantly, PPQ was previously considered fast-acting according to the classical PRR assay conducted with the drug-sensitive *P. falciparum* isolate NF54 and its clone 3D7 (see **Figure 1B**, and Sanz et al. (2012)). In the Cambodian field isolate RF12, in contrast, the compound was classified as slow-acting or even inactive.

In that regard, it was particularly striking that the potency shift (i.e. IC_50_ shift between drug-sensitive and -resistant parasites) was well below the threshold for drug resistance (defined as a shift ≥ 5 (Duffey et al., 2021) or even ≥ 20 (Ding et al., 2012)), indicating that a shift in IC_50_ is not sufficient to evaluate drug susceptibility. Growth inhibition assays conducted with PPQ-resistant parasites in prior studies did also not result in a substantial IC_50_ shift (Leang et al., 2013; Lim et al., 2013; Saunders et al., 2014; Bopp et al., 2018). Yet, the plateau towards higher drug concentrations in dose-response curves might be a first indicator of reduced drug susceptibility. Whether this holds true beyond PPQ remains to be validated in follow-up studies with additional field isolates and chemotypes. Still, minor plateaus may be easily overlooked, especially when the background signal of the underlying readout method is high. Therefore, we argue that viability assays with culture-adapted, drug-resistant field isolates may serve as complementary tool to evaluate the potency of selected, advanced candidates.

The classical PRR assay provides relevant pharmacodynamic parameters, such as the PRR or the *E_max_*, that shed light on the *in vitro* killing kinetics of a compound in drug-sensitive parasites; these can be used for downstream PK/PD analysis (Wicha et al., 2022; Walz et al., 2023), e.g. to predict the effective dose of single drugs or drug combinations in humans. However, our data suggest that solely relying on pharmacodynamic data from a single, drug-sensitive parasite strain may be insufficient, potentially resulting in overestimation of the drug effect in the field setting. Instead, we recommend to assess the pharmacodynamics of advanced drug candidates in the context of an *in vitro* PRR assay on a representative panel of drug-resistant field isolates, similar to the one deployed for cross-resistance testing in growth inhibition assays (Chugh et al., 2015; Duffey et al., 2021). This might result in more accurate predictions of drug efficacy in actual malaria patients, ultimately reducing the risk of late withdrawal of drug candidates (and concomitant financial losses and ethical concerns), and hence streamlining the drug development process.

*In vitro* washout assays other than the PRR assay, such as the ring-stage survival assay (RSA_0-3h_) for artemisinin derivatives (Witkowski et al., 2013; Walz et al., 2019) and the PPQ survival assay (PSA) (Duru et al., 2015), are valuable tools to measure the level of drug resistance in a short amount of time. However, their use is tailored to a specific drug, i.e. to mimic the pharmacokinetic profile (drug exposure and concentration observed in patients), with the intention to monitor the antimalarial efficacy of drugs that are already approved and routinely deployed in the field. The *in vitro* PRR assay, in contrast, allows predicting the risk of drug resistance of diverse preclinical candidates irrespective of their chemotype and pharmacokinetic profile.

The usefulness of parasite viability assays to assess drug resistance *in vivo* was also probed before. In contrast to the conventional measure of *in vivo* drug activity (i.e. parasite clearance (White, 2011)), parasite viability differentiates between viable and nonviable parasite populations in a sample (Rebelo et al., 2020; Radohery et al., 2022a; Radohery et al., 2022b). Not doing so might result in underestimation of drug efficacy as demonstrated for artesunate in studies conducted in mice and humans (Rebelo et al., 2020; Radohery et al., 2022b). Since *in vivo* viability assays provide a more sensitive estimate of drug activity than parasite clearance, they are expected to spot signs of drug resistance earlier. Using a mathematical model, Hastings et al. found that *in vivo* parasite clearance is highly insensitive at detecting drug resistance and does so only if resistance is sufficiently strong, as observed with *kelch13* mutations (Hastings et al., 2015). Later, Rebelo et al. corroborated this theory by showing that volunteers infected with artemisinin-resistant parasites had a 2-fold longer parasite clearance half-life than volunteers infected with artemisinin-sensitive parasites, whereas the difference in half-lives was 12-fold when parasite viability was measured instead (Rebelo et al., 2020).

In conclusion, assessing parasite viability following treatment in culture-adapted field isolates has the potential to improve our understanding of antimalarial drug resistance. It will likely result in more accurate predictions of drug efficacy, thereby guiding resistance monitoring in the field and aiding decision-making in preclinical and clinical drug development. In addition, measuring parasite viability in a representative panel of field isolates might help to detect drug resistance earlier, and therefore would allow faster switching of national treatment policies, ultimately preventing the spread of drug resistance.

## Data availability statement

The raw data supporting the conclusions of this article will be made available by the authors, without undue reservation.

## Ethics statement

The manuscript presents research on animals that do not require ethical approval for their study.

## Author contributions

AW: Conceptualization, Formal analysis, Methodology, Validation, Visualization, Writing – original draft, Writing – review & editing. SS: Investigation, Validation, Writing – review & editing. CS: Investigation, Validation, Writing – review & editing. BT: Formal analysis, Visualization, Writing – review & editing. PM: Resources, Supervision, Writing – review & editing. SW: Conceptualization, Funding acquisition, Methodology, Project administration, Resources, Supervision, Writing – review & editing.
