# Supplementary material for: Incomplete Plasmodium falciparum growth inhibition following piperaquine treatment translates into increased parasite viability in the in vitro parasite reduction ratio assay
Source: Front Cell Infect Microbiol. 2024 Apr 30;14:1396786. doi: 10.3389/fcimb.2024.1396786 (PMC11091375; doi:10.3389/fcimb.2024.1396786)
Supplement: Supplementary file 1 [file DataSheet_1.docx]

Supplementary Material

# Supplementary Figures


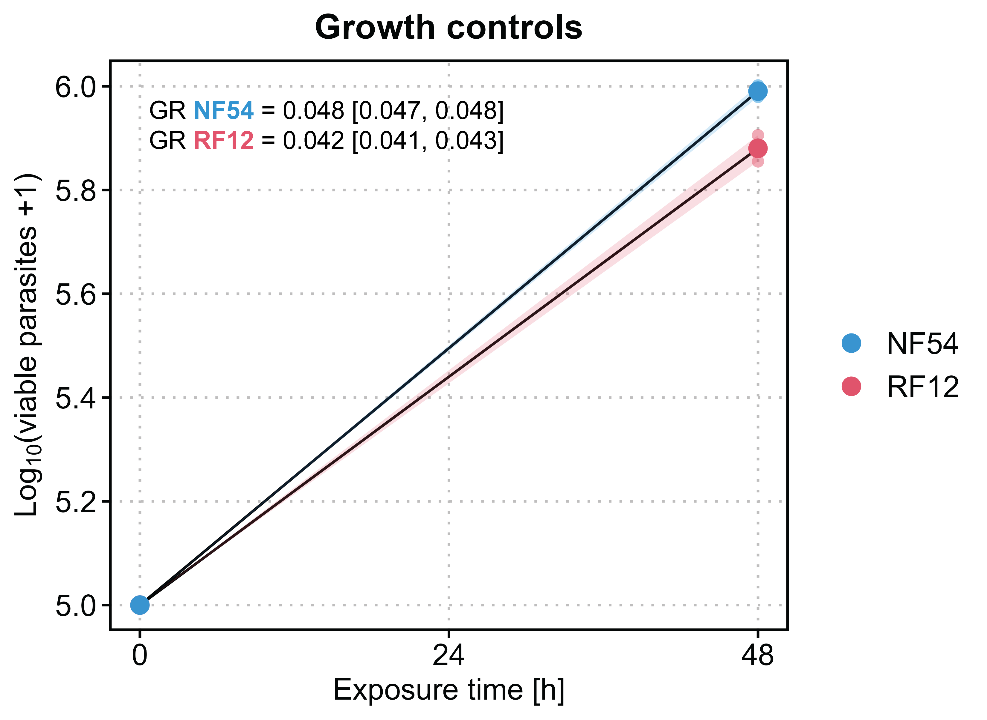


**Supplementary Figure 1.** Untreated growth controls of the parasite reduction ratio (PRR) assays conducted with *P. falciparum* NF54 (blue) and RF12 (red). Large dots are the means of n = 2 biological replicates, small dots are the means of individual biological replicates, each consisting of n = 3 technical replicates. The shaded area represents the range between the two biological replicates. GR, growth rate (in natural logarithm scale).
