# Supplementary material for: Incomplete Plasmodium falciparum growth inhibition following piperaquine treatment translates into increased parasite viability in the in vitro parasite reduction ratio assay
Source: Front Cell Infect Microbiol. 2024 Apr 30;14:1396786. doi: 10.3389/fcimb.2024.1396786 (PMC11091375; doi:10.3389/fcimb.2024.1396786)
Supplement: Supplementary file 2 [file DataSheet_2.pdf]

Statistical Appendix

1 Bayesian hierarchical dose-response analysis

The statistical model comprises two main elements: the dose-response curves, which describe the location (central tendency or mean, if it exists) of the response and the errors that capture the departures of the individual observations around this location.

$$Y_{ij} = f_i(x_{ij}, \boldsymbol{\vartheta}_i) + \varepsilon_{ij}, \tag{1}$$

with $\boldsymbol{\vartheta}_i$ as the vector of parameters of a given biological replicate.

The observations across the $i = 1 \dots N$ biological replicates are not exchangeable, but within the same biological replicate, different technical replicates are *assumed* to be exchangeable conditionally on the dose, so $j = 1 \dots n_i$ indexes both the technical replicates and the applied doses.

The various strain (*RF12* and *NF54*) and drug (chloroquine diphosphate [*cq*] and piperaquine tetraphosphate [*ppq*]) combinations are analyzed separately.

1.1 Two-parameter and three-parameter dose-response curves

For a given biological replicate i, the dose-response relationship is captured by either the two-parameter ($\boldsymbol{\vartheta}_i = (\alpha_i, \beta_i)^\top$),

$$f_i(x_{ij}, \boldsymbol{\vartheta}_i) = 100 \left(1 - \frac{1}{1 + \exp\left\{\alpha_i \left[\log(\beta_i) - \log(x_{ij})\right]\right\}}\right),$$

or the three-parameter ($\boldsymbol{\vartheta}_i = (\alpha_i, \beta_i, \gamma_i)^\top$) log-logistic model,

$$f_i(x_{ij}, \boldsymbol{\vartheta}_i) = 100 \left(1 - \frac{\gamma_i}{1 + \exp\left\{\alpha_i \left[\log(\beta_i) - \log(x_{ij})\right]\right\}}\right),$$

where α_i is the Hill slope, β_i denotes the IC_{50}, and $100(1 - \gamma_i)$ is the plateau level, to which the response converges as the dose increases.

1.2 Population-level parameters, grouping

The dose-response curves associated with individual biological replicates are allowed to have their own (either three or two) parameters. These parameters are assumed to vary according

to the normal distribution around the population-level parameters on either the logistic or the *logit* scale,

$$\begin{aligned}
\log(\alpha_i) &= \mu_\alpha + \sigma_\alpha \theta_i^\alpha, \qquad && \theta_i^\alpha \sim \mathcal{N}(0,1) \\
\log(\beta_i) &= \mu_\beta + \sigma_\beta \theta_i^\beta, \qquad && \theta_i^\beta \sim \mathcal{N}(0,1) \\
\log\left(\frac{\gamma_i}{1-\gamma_i}\right) &= \mu_\gamma + \sigma_\gamma \theta_i^\gamma, \qquad && \theta_i^\gamma \sim \mathcal{N}(0,1),
\end{aligned}$$

applying a non-centered parameterization (Betancourt and Girolami, 2015).

1.3 Error structure

The error captures the departures of the individual measurements from the central tendency, which represents the underlying biological mechanism.

The errors are assumed to be heteroskedastic: Their scale consists of an absolute element and a relative element, which is proportional to the mean (or the central tendency):

$$\begin{aligned}
\varepsilon_{ij}(\mu_{ij}, \sigma_a, \sigma_r) &= \sqrt{\sigma_a^2 + \sigma_r^2 \mu_{ij}^2}\,\varepsilon_{ij}^\star \\
\mu_{ij} &= \frac{f_i(x_{ij}, \boldsymbol{\vartheta}_i)}{100}
\end{aligned}$$

The use of heteroskedastic errors is motivated by the fact that the outcome, i.e., relative growth, is calculated as a ratio of counts, and both ratios and counts tend to be heteroskedastic. This property can be simply verified by looking at the raw data (see Figure 3). A heteroskedastic error structure in a Bayesian dose-response analytic context was also proposed by Hennessey et al. (2010) and Rønneberg et al. (2021).

$\varepsilon_{ij}^\star$ is i.i.d. and we consider two cases: Normally distributed errors, i.e., $\varepsilon_{ij}^\star \sim \mathcal{N}(0,1)$, and heavy-tailed (Student t-distributed) errors, i.e., $\varepsilon_{ij}^\star \sim t_\nu$, where ν is an additional degrees-of-freedom parameter to be estimated from the data. This latter extension might be useful for adapting our model to outliers and motivated by the fact that, in many cases, ratio distributions tend to be heavy-tailed (Díaz-Francés and Rubio, 2013). Patel et al. (2012) also used t-distributed errors in their dose-response analysis. It is worth mentioning, that the Gaussian error case is nested within the t-distributed error model, i.e., $\varepsilon_{ij}^\star$ becomes standard normal as $\nu \to \infty$.

1.4 Priors

The following prior distributions were defined for our models:

$$\begin{aligned}
\mu_\alpha &\sim \mathcal{N}(\log(5), 2)\\
\mu_\beta &\sim \mathcal{N}(\log(80), 3)\\
\mu_\gamma &\sim \mathcal{N}(\text{logit}(0.85), 1)\\
\sigma_\alpha &\sim \text{Half-Normal}(0, 0.5)\\
\sigma_\beta &\sim \text{Half-Normal}(0, 1)\\
\sigma_\gamma &\sim \text{Half-Normal}(0, 0.5)\\
\sigma_a &\sim \text{Half-Normal}(0, 5)\\
\sigma_r &\sim \text{Half-Normal}(0, 100)\\
\nu &\sim \Gamma(2, 0.1)
\end{aligned}$$

The resulting prior predictive distributions (Gabry et al., 2019) of the two- and three-parameter models, with Gaussian and Student t-distributed errors, respectively, are presented in Figure 1, along with the observed response values.

1.5 Estimation and inference

The inference is based on samples from the posterior distribution of the model parameters generated with the Hamiltonian Monte Carlo algorithm using Stan (Carpenter et al., 2017). The performance of the sampling procedure was checked by typical diagnostic measures (Betancourt, 2018).

2 Results

We considered both two-parameter and three-parameter dose-response models for each strain-drug combination. The latter model variant allows for the response to plateau at a nonzero level when high concentrations of the compound are applied. The most appropriate model for each drug-strain combination was selected using cross-validation measures (see Section 2.1). These measures strongly suggest the presence of a plateau in the dose-response relationship of *RF12* with piperaquine, whereas the two-parameter models were sufficient to capture the dose-response curves in the other strain-drug combinations (*NF54* with both drugs and *RF12* with chloroquine diphosphate). Figure 3 presents the population-level dose-response curves along with the corresponding 95%, 90% and 50% credible intervals of the posterior predictive distributions. These intervals are expected to contain the dose-response curves from an experiment on a new biological replicate with 0.95, 0.9 and 0.5 probability, respectively. Medians and 90% credible intervals of the population-level parameters of the hierarchical models are compared in Table 1. We also derive estimates for the probability of 90% or higher inhibition, at given concentration levels, for each strain-drug combination and all model variants considered (Figure 4). These results also highlight that dose-response relationship of piperaquine for *RF12* stands out in that incomplete inhibition is much more likely with this strain-drug combination.

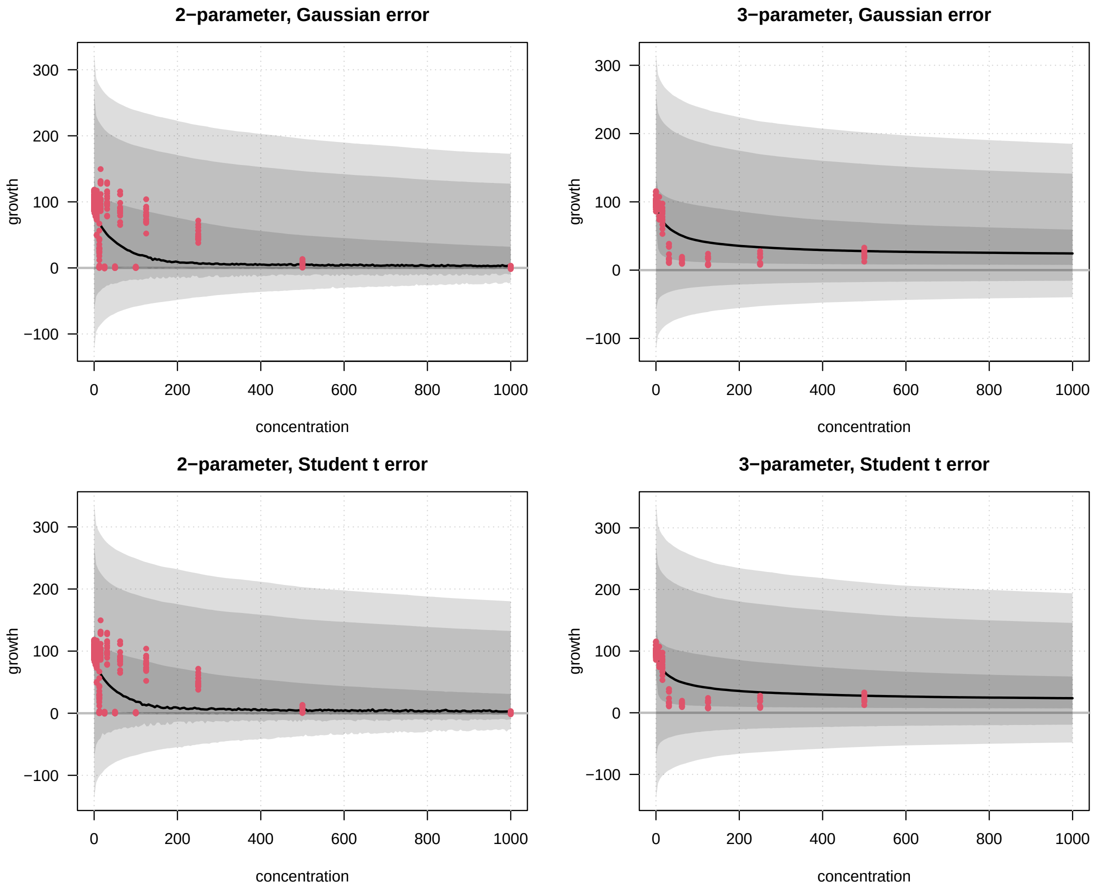

Figure 1: Prior predictive distributions of the response implied by the four model variants. The shaded areas denote the 95%, 90% and 50% predictive intervals, respectively, while the solid black lines are the medians of the prior predictive distributions. The red dots present the observations: *RF12-ppq* in the case of the three-parameter models, and the rest in the two-parameter cases.

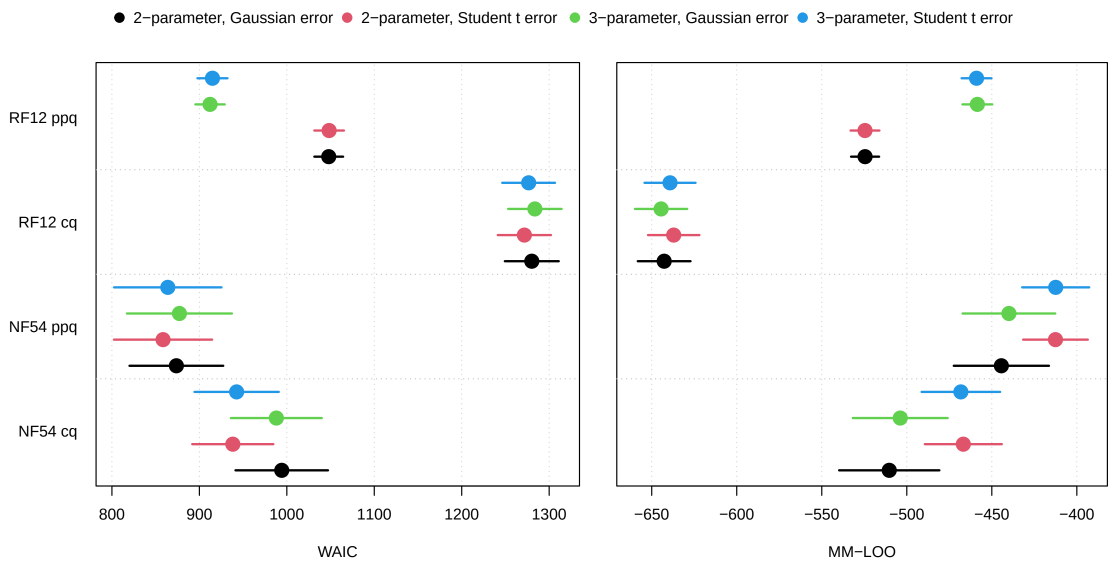

Figure 2: Model comparison. The four model variants are compared based on the widely applicable information criterion (WAIC, *left panel*) and moment-matched leave-one-out cross validation (MM-LOO, *right panel*).

2.1 Model comparisons

We fitted all four model variants presented in Section 1 to the four datasets representing each strain-drug combination, and compared the results based on leave-one-out cross-validation (LOO-CV, Vehtari et al., 2017). Figure 2 shows the widely applicable information criterion (WAIC, Vehtari et al., 2017) and the moment-matched LOO (Paananen et al., 2021). Based on these measures, two patterns can be discerned: The three-parameter model only has an advantage in the case of *RF12-ppq*, and non-normal errors result in slightly better fitting models, especially in the case of *NF54*.

2.2 Posterior predictive distributions

Our primary interest is to estimate the *population-level* response distribution conditionally on the dose level, which represents the distribution of the dose-reponse curve in a new experiment for a new biological replicate. Building on the results in Section 2.1, we primarily focus on the model versions with Student t-distributed errors. For the strain-drug combination *RF12-ppq*, we use the three-parameter dose-response model, for the others (*NF54-cq*, *NF54-ppq* and *RF12-cq*), we present the two-parameter version.

Figure 3 shows the posterior predictive distributions of the four hierarchical dose-response models fitted to the different strain-drug combinations along with the corresponding observations.

Figure 4 presents the posterior probabilities of observing 90% or higher inhibition, as a function of the concentration, i.e., the probability $\mathbb{P}(Y_{ij} \leq 0.1 \mid x_{ij})$, in the case of the four strain-drug combinations. The plots compare all model variants, and confirm that the three-parameter model is more adequate in the case of *RF12-ppq*.

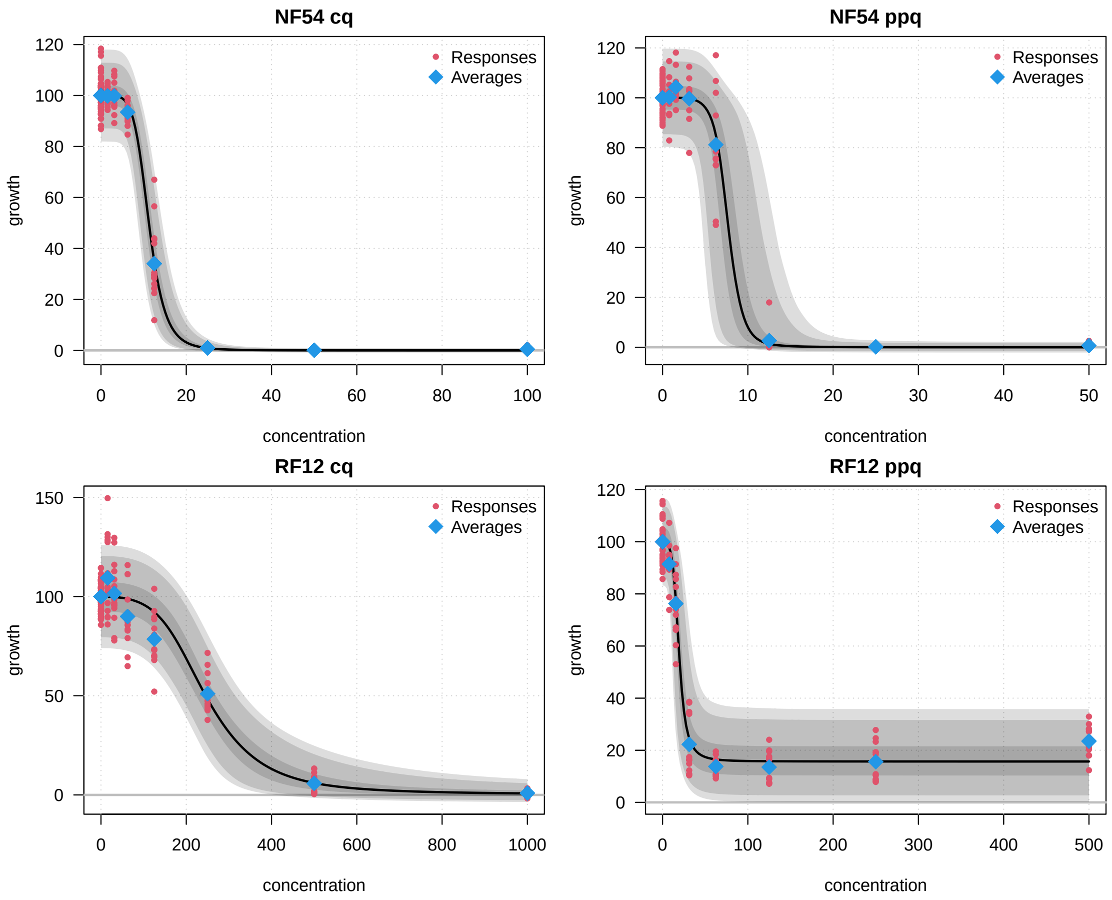

Figure 3: Posterior predictive distributions of the response at different concentration levels. The shaded areas denote the 95%, 90% and 50% predictive intervals, respectively, while the solid black lines are the medians of the posterior predictive distributions.

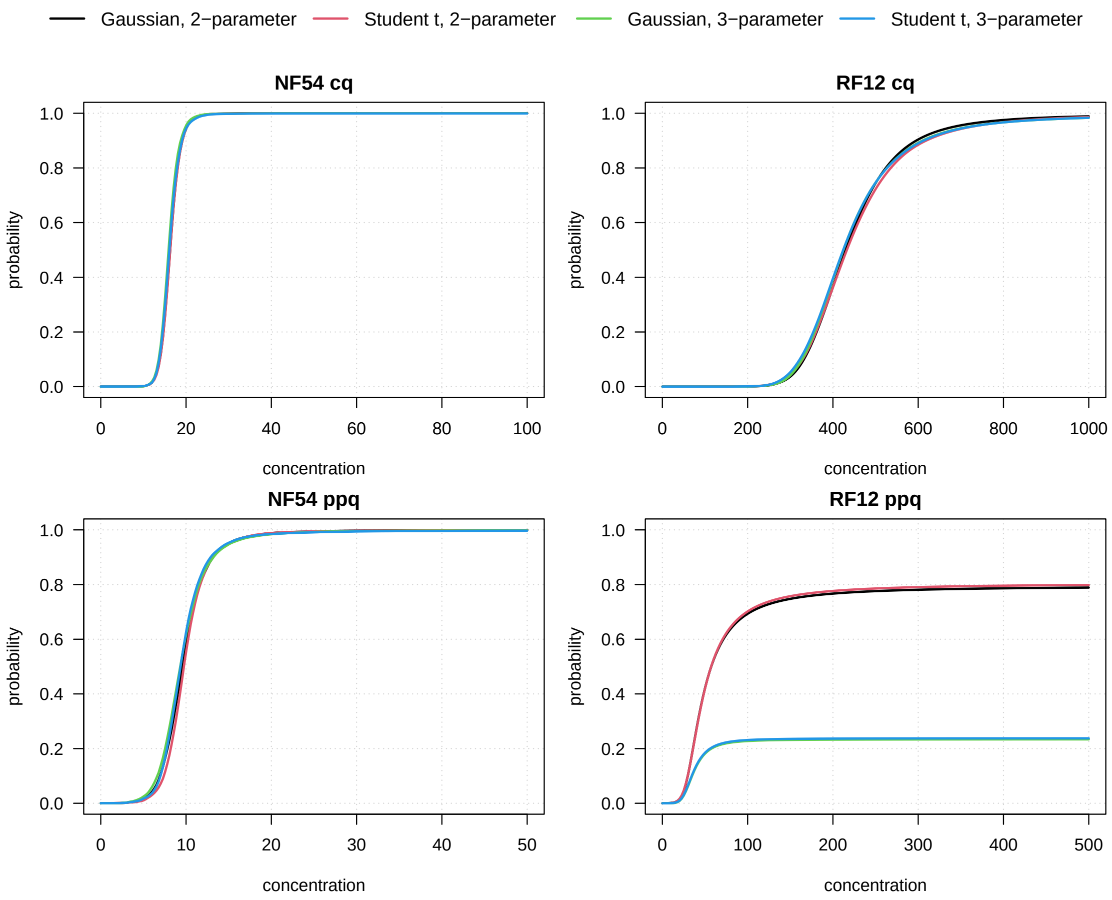

Figure 4: Posterior probability of 90% or larger inhibition.

Table 1: Medians and 90% equitailed credible intervals of the population-level parameters of the hierarchical dose-response models. The two-parameter models fix the plateau at 0, i.e., no plateau effect is allowed in these models. In the case of the three-parameter model, only a 95% *one-sided* credible interval is reported for the IC_{90} level (cf. Figure 5).

		NF54	RF12
cq	Hill slope (α_i)	5.97 (4.80 – 7.64)	3.95 (2.23 – 7.32)
	IC_{50} (β_i)	11.26 (9.31 – 13.64)	246.71 (199.03 – 308.20)
	Plateau ($100(1-\gamma_i)$)	0	0
	IC_{90}	16.29 (13.02 – 20.52)	430.46 (295.12 – 753.61)
ppq	Hill slope (α_i)	9.28 (6.21 – 16.84)	4.98 (3.36 – 8.39)
	IC_{50} (β_i)	7.66 (5.30 – 11.43)	19.25 (12.47 – 29.65)
	Plateau ($100(1-\gamma_i)$)	0	15.31 (7.60 – 28.09)
	IC_{90}	9.74 (6.59 – 15.08)	(26.95 –)

2.3 Parameters

Table 1 presents the population-level parameter distributions of the two- and three-parameter hierarchical dose-response models with their medians and 90% equitailed credible intervals. These intervals contain the parameters of the dose-response curve from an experiment involving a new biological replicate with a probability of 0.9. The two-parameter models fix the γ_i parameter at 1, i.e., there is no plateau effect in those models ($100(1-\gamma_i) = 0$).

The 90% inhibitory concentration levels (IC_{90}) in Table 1 are calculated by inverting the dose-response relationship and solving Equation 1 for a relative growth level of 10%. In the case of the three-parameter model, the posterior distribution of this parameter has a point mass on infinity, i.e., not all model parameter values result in curves that cross the 10% relative growth level. This phenomenon is demonstrated in Figure 5. For this reason, Table 1 reports a 95% one-sided credible interval for the *RF12-ppq* strain-drug combination.

2.4 Model checking

We performed posterior predictive checks to evaluate the fitted models (Gabry et al., 2019). Figure 6 compares the kernel density estimates of the observed data with estimates for simulated datasets from the posterior predictive distributions of the hierarchical dose-response models. The results confirm that fitted models capture the main properties of the marginal outcome distributions.

The calibration of the predictions is assessed using LOO probability integral transforms (Gabry et al., 2019) in Figure 7. Under a well-calibrated model, the cross-validated probability integral transforms are asymptotically uniformly distributed. Only slight deviations are observed in the case of *RF12-ppq*. In the next subsection, we investigate this phenomenon further.

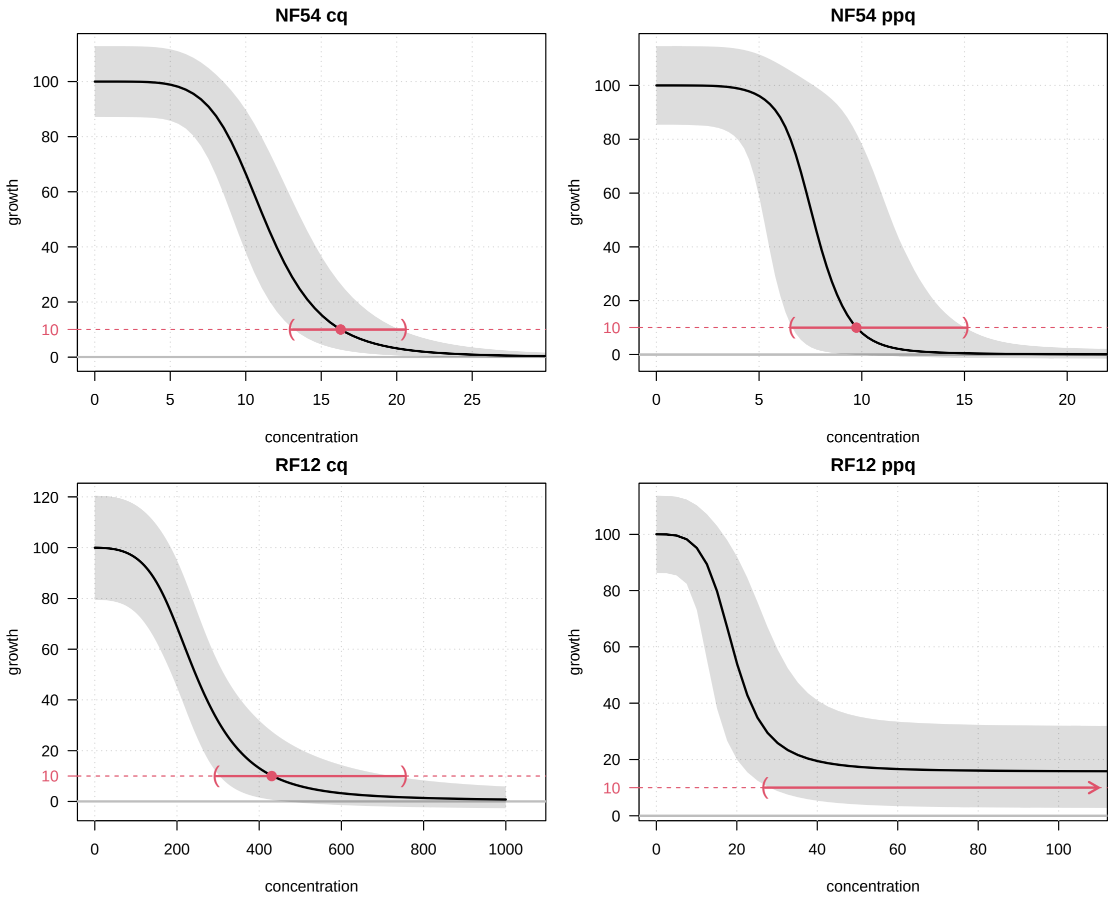

Figure 5: Point estimates (red points) and credible intervals (red segments) for IC_{90}. The grey bands represent the 90% prediction intervals for the dose-response curves (see also Figure 3). In the case of the three-parameter model (bottom-right panel), the posterior of IC_{90} has a point mass at infinity and only a one-sided credible interval is calculated.

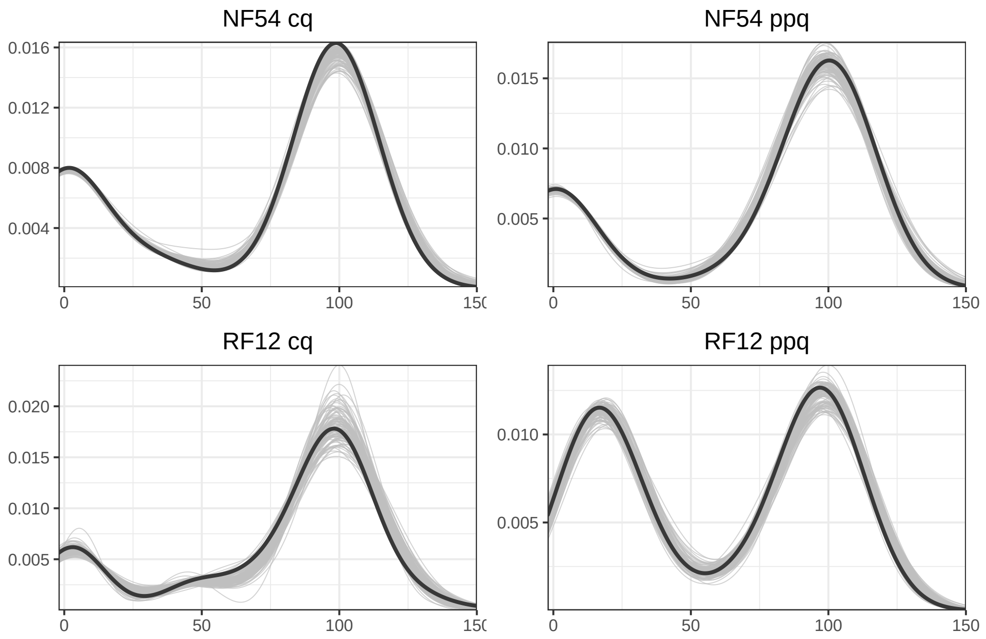

Figure 6: Kernel density estimates of the observed data (black curves), and density estimates for 100 datasets generated from the posterior predictive distributions (grey curves).

Table 2: Medians and 90% equitailed credible intervals of the population-level parameters of the hierarchical dose-response model for *RF12-ppq* fitted with and without removing the observations with the highest concentration levels.

	Full sample	Restricted sample
Hill slope (α_i)	4.98 (3.36 – 8.39)	5.41 (2.84 – 12.39)
IC_{50} (β_i)	19.25 (12.47 – 29.65)	19.83 (12.30 – 32.03)
Plateau ($100(1-\gamma_i)$)	15.31 (7.60 – 28.09)	12.76 (5.55 – 26.28)

2.5 Sensitivity check

In Section 2.4, we observed a mild miscalibration of the three-parameter hierarchical model for *RF12-ppq* (see the bottom-right segment of Figure 7). This discrepancy might be due to the increasing tendency of the response values at the highest dose level in the data, which is at odds with the assumed monotonic decreasing relationship represented by the log-logistic dose-response curve. Note, however, that our model is still compatible with actual non-decreasing observations because we assume that the responses are always observed with an error (ε_{ij} in Equation 1). Nevertheless, as a robustness check, we will now asses how our estimates change when we remove the observations corresponding to 500 nM doses.

As Figure 8 and Table 2 suggest, restricting our sample increases the uncertainty around the estimates, but the main qualitative properties of the results and our conclusions remain unchanged.

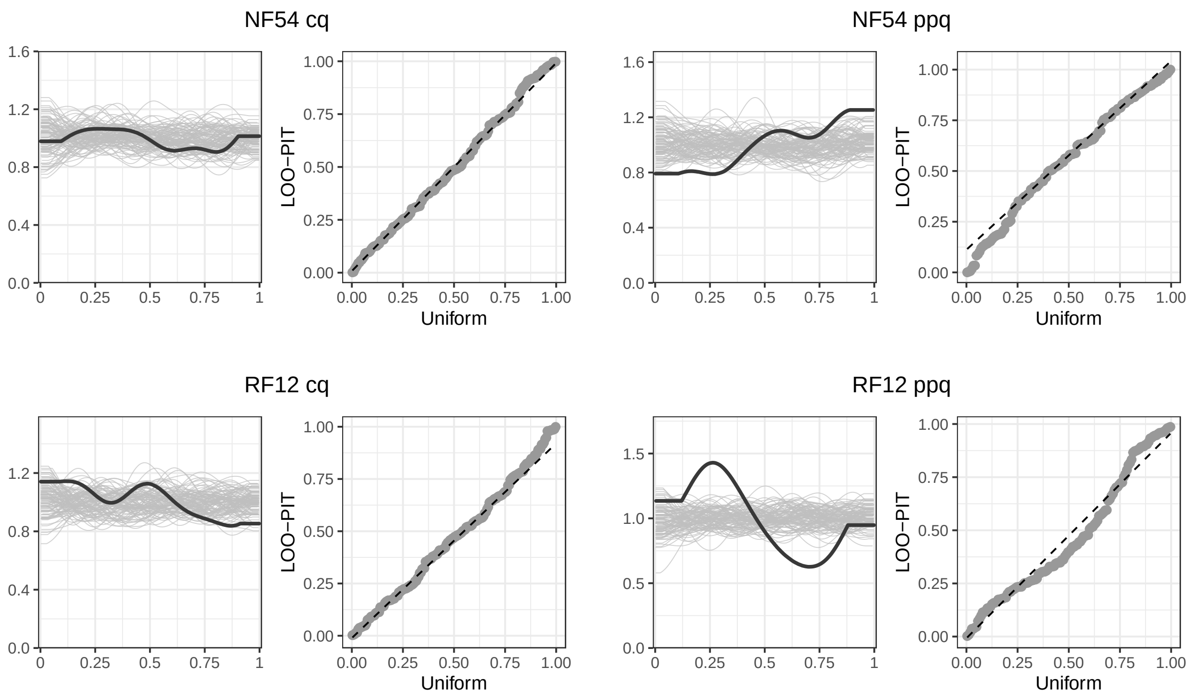

Figure 7: Graphical checks of the LOO-CV probability integral transforms. *Left blocks:* Density calculated from the LOO probability integral transfroms (black curves) along with simulations from the standard uniform distribution (grey curves). *Right blocks:* Empirical quantiles of the LOO probability integral transforms against the theoretical quantiles of the standard uniform distribution.

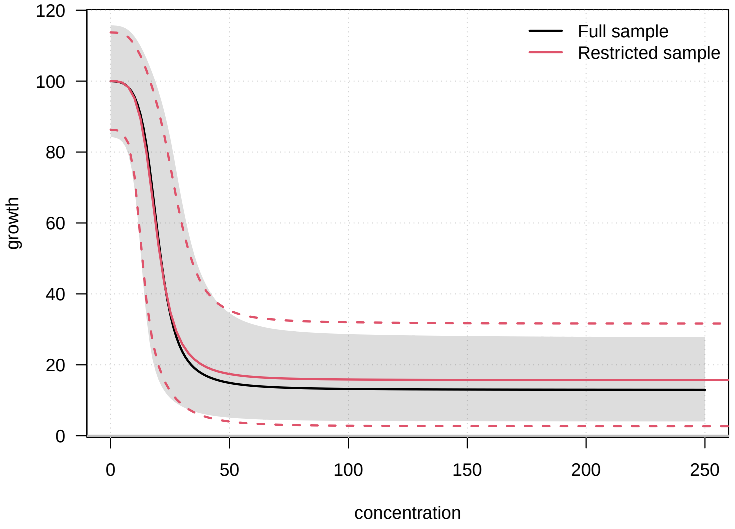

Figure 8: Medians and 90% credible intervals of the posterior predictive distributions for *RF12-ppq* with and without removing the observations with the highest concentration levels.

Computational details

The posterior sampling was carried out using the Hamiltonian Monte Carlo algorithm implemented by Stan (Carpenter et al., 2017) and using the interface provided by the **cmdstanr** package in R (Gabry et al., 2023). Leave-one-out cross-validations were calculated with the help of the **loo** package (Vehtari et al., 2023), while the plots for model checking were generated with the **bayesplot** package (Gabry and Mahr, 2022).

```
R> sessionInfo()

R version 4.3.2 (2023-10-31)
Platform: x86_64-pc-linux-gnu (64-bit)
Running under: Ubuntu 22.04.3 LTS

Matrix products: default
BLAS:   /usr/lib/x86_64-linux-gnu/blas/libblas.so.3.10.0
LAPACK: /usr/lib/x86_64-linux-gnu/lapack/liblapack.so.3.10.0

locale:
 [1] LC_CTYPE=C.UTF-8       LC_NUMERIC=C           LC_TIME=C.UTF-8
 [4] LC_COLLATE=C.UTF-8     LC_MONETARY=C.UTF-8    LC_MESSAGES=C.UTF-8
 [7] LC_PAPER=C.UTF-8       LC_NAME=C              LC_ADDRESS=C
[10] LC_TELEPHONE=C         LC_MEASUREMENT=C.UTF-8 LC_IDENTIFICATION=C

time zone: Europe/Zurich
tzcode source: system (glibc)

attached base packages:
[1] stats     graphics  grDevices datasets  utils     methods   base

other attached packages:
[1] xtable_1.8-4     patchwork_1.2.0  ggplot2_3.4.4    bayesplot_1.10.0
[5] loo_2.6.0        cmdstanr_0.6.1

loaded via a namespace (and not attached):
 [1] gtable_0.3.4         jsonlite_1.8.8       highr_0.10
 [4] dplyr_1.1.4          compiler_4.3.2       tidyselect_1.2.0
 [7] bspm_0.5.5           parallel_4.3.2       scales_1.3.0
[10] R6_2.5.1             generics_0.1.3       distributional_0.3.2
[13] knitr_1.45           backports_1.4.1      checkmate_2.3.1
[16] tibble_3.2.1         munsell_0.5.0        pillar_1.9.0
[19] posterior_1.5.0      rlang_1.1.3          utf8_1.2.4
[22] xfun_0.41            cli_3.6.2            withr_2.5.2
[25] magrittr_2.0.3       ps_1.7.5             digest_0.6.34
[28] grid_4.3.2           processx_3.8.3       lifecycle_1.0.4
[31] vctrs_0.6.5          evaluate_0.23        glue_1.7.0
[34] tensorA_0.36.2.1     farver_2.1.1         abind_1.4-5
[37] fansi_1.0.6          colorspace_2.1-0     matrixStats_1.2.0
[40] tools_4.3.2          pkgconfig_2.0.3
```

Date: `Tue Mar 5 12:45:28 2024`.

References

Michael Betancourt. A conceptual introduction to Hamiltonian Monte Carlo, 2018. arXiv:1701.02434 [stat.ME].

Michael Betancourt and Mark Girolami. Hamiltonian Monte Carlo for hierarchical models. In Satyanshu K. Upadhyay, Umesh Singh, Dipak K. Dey, and Appaia Loganathan, editors, *Current trends in Bayesian methodology with applications*, chapter 4, pages 79–102. CRC Press Boca Raton, FL, 2015.

Bob Carpenter, Andrew Gelman, Matthew D. Hoffman, Daniel Lee, Ben Goodrich, Michael Betancourt, Marcus Brubaker, Jiqiang Guo, Peter Li, and Allen Riddell. Stan: A probabilistic programming language. *Journal of Statistical Software*, 76(1):1–32, 2017. doi:10.18637/jss.v076.i01.

Eloísa Díaz-Francés and Francisco J. Rubio. On the existence of a normal approximation to the distribution of the ratio of two independent normal random variables. *Statistical Papers*, 54(2):309–323, May 2013. doi:10.1007/s00362-012-0429-2.

Jonah Gabry and Tristan Mahr. **bayesplot**: Plotting for Bayesian models, 2022. URL `https://mc-stan.org/bayesplot/`. R package version 1.10.0.

Jonah Gabry, Daniel Simpson, Aki Vehtari, Michael Betancourt, and Andrew Gelman. Visualization in Bayesian workflow. *Journal of the Royal Statistical Society: Series A (Statistics in Society)*, 182(2):389–402, 2019. doi:10.1111/rssa.12378.

Jonah Gabry, Rok Češnovar, and Andrew Johnson. ***cmdstanr**: R Interface to 'CmdStan'*, 2023. URL `https://mc-stan.org/cmdstanr/`. R package version 0.6.1.

Violeta G. Hennessey, Gary L. Rosner, Robert C. Bast Jr, and Min-Yu Chen. A Bayesian approach to dose-response assessment and synergy and its application to in vitro dose-response studies. *Biometrics*, 66(4):1275–1283, 2010. doi:10.1111/j.1541-0420.2010.01403.x.

Topi Paananen, Juho Piironen, Paul-Christian Bürkner, and Aki Vehtari. Implicitly adaptive importance sampling. *Statistics and Computing*, 31(2):16, 2021. doi:10.1007/s11222-020-09982-2.

Trina Patel, Donatello Telesca, Saji George, and André E. Nel. Toxicity profiling of engineered nanomaterials via multivariate dose-response surface modeling. *The Annals of Applied Statistics*, 6(4):1707–1729, 2012. doi:10.1214/12-AOAS563.

Leiv Rønneberg, Andrea Cremaschi, Robert Hanes, Jorrit M Enserink, and Manuela Zucknick. **bayesynergy**: Flexible Bayesian modelling of synergistic interaction effects in in vitro drug combination experiments. *Briefings in Bioinformatics*, 22(6):bbab251, 2021. doi:10.1093/bib/bbab251.

Aki Vehtari, Andrew Gelman, and Jonah Gabry. Practical Bayesian model evaluation using leave-one-out cross-validation and WAIC. *Statistics and Computing*, 27(5):1413–1432, 2017. doi:10.1007/s11222-016-9696-4.

Aki Vehtari, Jonah Gabry, Mans Magnusson, Yuling Yao, Paul-Christian Brürkner, Topi Paananen, and Andrew Gelman. **loo**: Efficient leave-one-out cross-validation and WAIC for Bayesian models, 2023. URL `https://mc-stan.org/loo/`. R package version 2.6.0.
